# A Means of Assessing Deep Learning-Based Detection of ICOS Protein Expression in Colon Cancer

**DOI:** 10.3390/cancers13153825

**Published:** 2021-07-29

**Authors:** Md Mostafa Kamal Sarker, Yasmine Makhlouf, Stephanie G. Craig, Matthew P. Humphries, Maurice Loughrey, Jacqueline A. James, Manuel Salto-Tellez, Paul O’Reilly, Perry Maxwell

**Affiliations:** 1 Precision Medicine Centre of Excellence, The Patrick G Johnston Centre for Cancer Research, Queen’s University Belfast, Belfast BT9 7AE, UK; m.sarker@qub.ac.uk (M.M.K.S.); y.makhlouf@qub.ac.uk (Y.M.); stephanie.craig@qub.ac.uk (S.G.C.); Matthew.Humphries2@nhs.net (M.P.H.); j.james@qub.ac.uk (J.A.J.); m.salto-tellez@qub.ac.uk (M.S.-T.); 2 Cellular Pathology, Belfast Health and Social Care Trust, Belfast City Hospital, Lisburn Road, Belfast BT9 7AB, UK; maurice.loughrey@belfasttrust.hscni.net; 3 Northern Ireland Biobank, The Patrick G Johnston Centre for Cancer Research, Queen’s University Belfast, Belfast BT9 7AE, UK; 4 Division of Molecular Pathology, The Institute of Cancer Research, Sutton SM2 5NG, UK; 5 Sonrai Analytics LTD, Lisburn Road, Belfast BT9 7BL, UK

**Keywords:** colorectal cancer, immunohistochemistry, biomarkers, ICOS, artificial intelligence, deep learning

## Abstract

**Simple Summary:**

In this study, we propose a general artificial intelligence (AI) based workflow for applying deep learning to the problem of cell identification in immunohistochemistry-stained slides as a basis for quantifying nuclear staining biomarkers. Our approach consists of two main parts: a simplified but robust annotation process, and the application of cell identification models. This results in an optimised process with a new user-friendly tool that can interact with other open-source software and assists pathologists and scientists in creating and exporting data for deep learning. We present a set of architectures for cell identification to quantify and analyse the trade-offs between different deep learning architectures, providing a more accurate and less time-consuming tool than using traditional methods. This approach can identify the best tool to deliver AI tools for clinical utility.

**Abstract:**

Biomarkers identify patient response to therapy. The potential immune-checkpoint biomarker, Inducible T-cell COStimulator (ICOS), expressed on regulating T-cell activation and involved in adaptive immune responses, is of great interest. We have previously shown that open-source software for digital pathology image analysis can be used to detect and quantify ICOS using cell detection algorithms based on traditional image processing techniques. Currently, artificial intelligence (AI) based on deep learning methods is significantly impacting the domain of digital pathology, including the quantification of biomarkers. In this study, we propose a general AI-based workflow for applying deep learning to the problem of cell segmentation/detection in IHC slides as a basis for quantifying nuclear staining biomarkers, such as ICOS. It consists of two main parts: a simplified but robust annotation process, and cell segmentation/detection models. This results in an optimised annotation process with a new user-friendly tool that can interact with1 other open-source software and assists pathologists and scientists in creating and exporting data for deep learning. We present a set of architectures for cell-based segmentation/detection to quantify and analyse the trade-offs between them, proving to be more accurate and less time consuming than traditional methods. This approach can identify the best tool to deliver the prognostic significance of ICOS protein expression.

## 1. Introduction

Colorectal cancer (CRC) is the third most deadly cancer with around 0.9 million deaths, with an estimated new-cases rise to 2.2 million annually and 1.1 million deaths by 2030 [1]. Molecular biomarkers, such as microsatellite instability, are an integral part of CRC diagnosis and can be used to inform the clinical management of the patient treatment pathway [2,3]. An emergent area of clinical practice is the use of immuno-oncology to treat patients who do not respond to conventional cytotoxic chemotherapy. Patient suitability to receive many of these novel therapeutic agents is based on the assessment of a companion biomarker, such as PD-L1 immunohistochemistry (IHC) [4]. Tissue-based biomarker analysis using IHC, retains spatial and cell-specific information, enabling accurate analysis of biomarker expression within the tumour microenvironment. The quantification of biomarker-expressing cells and their localization can then be assessed by a pathologist in order to provide prognostic and predictive patient information [5,6]. In the research setting, multiple tumour samples can be tested with IHC using tissue microarrays (TMA), as a means of high-volume throughput biomarker analysis [7,8]. However, manual assessment of TMA IHC analysis is slow, subjective, and not suitable for investigating large numbers of biomarker-expressing cells [9,10].

Computer-assisted image analysis systems can facilitate large-scale quantitative analysis of IHC on TMAs. Several studies have now been published on the benefit of computer-assisted quantitative cell count analyses [11] and automated tissue cell segmentation [12] versus manual assessment [13]. Recently, deep learning methods in artificial intelligence (AI) were introduced to the domain of digital image analysis in pathology images for nuclei detection [14], mitosis detection [15], growth pattern classification [16], lymphocyte detection [17] and patient stratification [18]. These encouraging initial methods are mostly focused on classifying tiles in whole-slide images and fluorescence images of cell lines [19,20] but to date are unable to reliably detect/segment biomarker-expressing cells on IHC-stained tissue images. In a previous study [21], we demonstrated the assessment of immune and immune-checkpoint biomarkers using a digital pathology image analysis system in stage II-IV CRC patients to evaluate the most useful biomarker or their combination to predict survival in CRC at diagnosis. In this study, we present an AI-enabled deep learning tool with the potential to deliver the prognostic significance of IHC biomarkers, through the creation of a robust, automated quantitative cell detection/segmentation system for the immune-checkpoint protein Inducible T-cell COStimulator (ICOS). Our proposed system will help diagnosticians and scientists to obtain accurate cell level information for nuclear-expressed proteins in the cancer microenvironment. Moreover, we introduce a robust and quantitative cell detection/segmentation system that can be utilized for other nuclear IHC biomarkers cell detection/segmentation challenges. 

## 2. Materials and Methods

**Figure 1:**
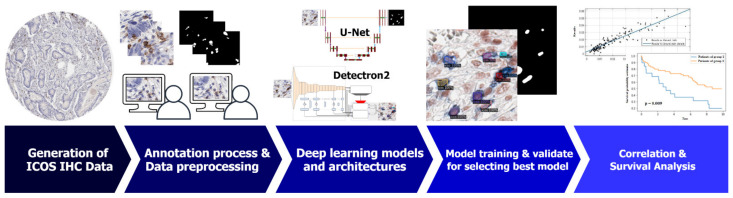
The step-by-step workflow: ICOS IHC data collection and annotated by expert pathologists; annotated data pre-processing for deep learning models; deep learning model selection and training; evaluating and selecting the best model; post-processing the best model outcomes and using them for ICOS correlation and survival analysis.

The step-by-step workflow of our study is presented in Figure 1. Initially, the ICOS IHC data are generated and annotated by expert pathologists. The annotated data are collected and prepared for the deep learning model training and validation. The best deep learning model is selected by testing all the models. The outcomes of the best model are post-processed and used in ICOS correlation (cell density estimation) and survival analysis, as detailed below. 

### 2.1. Generation of ICOS IHC Data

Comparison of deep learning models to detect ICOS protein expression was conducted in a representative population, a stage II and stage III colon adenocarcinoma cohort (Epi700), which was described previously [22]. The digital images used were generated by the Northern Ireland Biobank under study number NIB15-0168. The ICOS IHC was produced under standardised operating procedures within the Queen’s University Belfast Precision Medicine Centre of Excellence and reported in [21]. Consultant pathologists (JJ and MST) agreed upon ICOS assay optimization prior to ICOS IHC staining. Briefly, ICOS IHC was conducted on formalin-fixed paraffin-embedded Epi700 tissue samples in tissue microarray (TMA) format. Tissue samples were taken in triplicate from the donor blocks as 1 mm diameter cores from regions identified as a central tumour by a consultant pathologist (MBL). TMAs were then sectioned at 4 m using a rotary microtome and dried overnight at 37 °C in preparation for staining. The Leica Bond RX automated immunostainer (Leica Biosystems, Newcastle, UK) was used to carry out ICOS IHC. IHC staining was conducted using an anti-ICOS antibody (Cell Signalling Technology, ICOS (D1L2TTM) rabbit monoclonal antibody, Clone D1K2T, Cat. No. 89601).

The antibody was diluted 1:400 using antibody diluent and incubated for 15 min on the tissue following heat-induced epitope retrieval (HIER) with ER2 for 20 min. Antibody binding was visualised with enhanced DAB chromogen (Leica Biosystems, Bond Polymer Refine Detection, Cat. No. DS9800 and Leica Biosystems, Bond DAB Enhancer, Cat. No. AR9432). All Epi700 ICOS IHC stained slides were scanned at 400× magnification using the Leica Aperio AT2 and made available for digital assessment. Open-source image analysis software QuPath v.0.1.234 was used to determine digital scoring of the ICOS IHC within each TMA core. Scanned TMA slides were imported and de-arrayed to separate individual cores within the image for digital image analysis [23]. Each core was given a unique identifier, which could be linked back to the clinicopathological data available for that patient. Invalid cores (no core or no tumour) were removed from the analysis. Simple tissue detection was performed, and all cores were re-annotated to remove undesirable objects that would affect the IHC scoring. Once identified, the TMA cores were annotated for training and validating the deep learning models, using the workflow proposed below. 

### 2.2. Annotation Process

**Figure 2:**
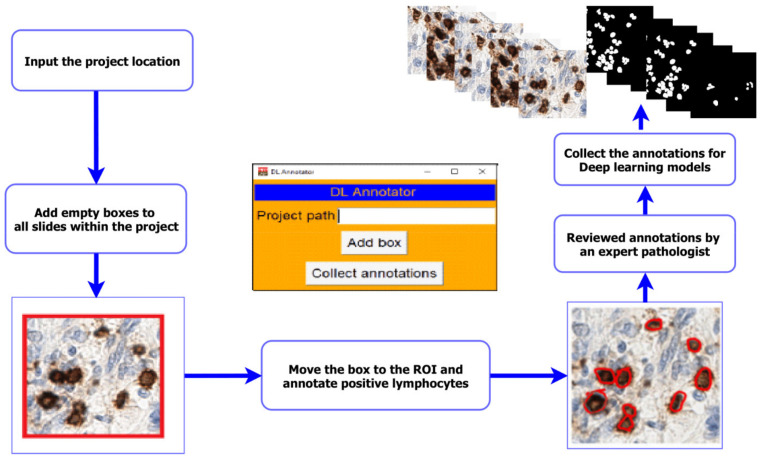
The pipeline of the annotation process. Creating projects for annotator and collection after completed annotations and review by the expert pathologist using the deep learning (DL) annotation tool.

The deep learning approach, which has generally shown the best performance in computational pathology algorithms, is that of supervised learning [24]. For supervised learning, there can be a requirement for large numbers of ground-truth annotations. Hence, the annotation process is an important part of training any model, but especially for the detailed annotation of nuclei required here, as it is a sometimes, tedious manual process for pathologists, and it can be difficult to track and extract the annotations in a format useful for deep learning. This often requires familiarity with scripting in order to build the final data set. To improve this task and make it accessible to any annotator, we propose a tool that simplifies this process and optimizes data collection. We designed an interface that interacts with the QuPath software [23] through simple plug-ins and with scripting in the background, from a user perspective. The workflow is as follows: the annotator specifies the project location and creates the regions of interest for annotation with a single click for all the images inside the project. Once the annotations are complete, the annotator can collect and extract the annotations with a single click. The final result is a set of folders containing all original patches and their corresponding masks. Figure 2 illustrates the overall process. Note that before any patch extraction, all slides are reviewed by a second reader (an expert pathologist). A pathologist may adjust the location where the boxes have been moved to and correct the cell boundaries. In this study, annotations were performed by PM and reviewed by MS-T.

### 2.3. Data Pre-Processing

IHC slides are very large with the 40× obj. magnification and not suitable to be fed into the input of deep learning models. Therefore, we create standard input patches (generated by the annotation process) that are suitable for training our models. We select the size of the patches to be 256 × 256 pixels, collect them with the annotation tool, and split the data set into the train, train-val, and test sets. The data set split ratio for the train, train-val, and test sets are 60%, 10%, and 30%, respectively.

Afterwards, we convert the format of the ground-truth images to binary in the case of the semantic segmentation model (U-Net [25]), and ms-coco format [26] in the case of the instance segmentation model (Detectron2 [27]). Note that we use only train and trainval to train and evaluate our deep learning models during training and keep the test set unseen for calculating the model performance.

### 2.4. Deep Learning Models and Architectures

**Figure 3:**
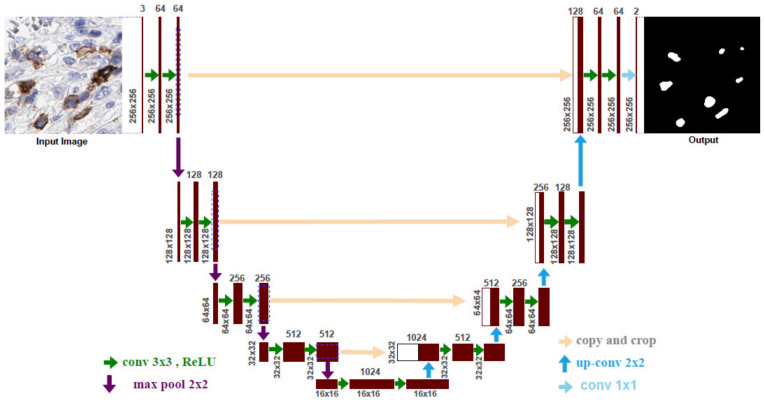
The detailed architecture of the U-Net has been modified from [25].

In this work, we present an assessment of the two deep learning approaches, semantic segmentation and instance segmentation-based models, for our ICOS biomarker cell detection/segmentation system. In the segmentation approach, U-Net [25] is a very popular deep learning model for the medical image segmentation domain. The U-Net model learns to segment the images in an end-to-end setting, which means a raw image as an input and a ready segmentation map as an output. The U-Net architecture consists of two paths: contraction and expansion. The contraction path (also known as the encoder) consists of a sequence of convolutions and max-pooling layers, which are used to capture the context in the input image. On the other hand, the expansion path (also known as the decoder) consists of a sequence of up-convolutions and concatenation with the corresponding high-resolution features from the contraction path that allows the creation of a high-resolution output segmentation map. The detailed architecture of U-Net is presented in Figure 3. Initially, we feed the U-Net with 256 × 256 × 3 input patches and process it with the contraction and expansion modules. The contraction module is composed of four contraction blocks and one bottleneck block. Each contraction block has two consecutive 3 × 3 convolutional layers followed by a rectified linear unit (ReLU). The process of convolution operations is to increase the channel-wise depth of the image. Four down-sample blocks with 2 × 2 max-pooling layers followed by a stride of 2 are applied after every contraction block. The down-sample blocks reduce the image size and double the number of feature channels for learning the complex structures effectively. The bottleneck block intercedes between the contraction module and the expansion module. It consists of 3 × 3 convolutional layers followed by a ReLU and 2 × 2 up convolution (up-conv) layer. The core contributions of the U-Net lie in the expansion module. The expansion module is also composed of four expansion blocks similar to the contraction module. Every block also consists of two 3 × 3 convolutional layers followed by a ReLU and 2 × 2 up-conv layer. After every contraction block, the feature maps are up sampled and attain the same size as the corresponding contraction block output to maintain harmony and concatenate it. This mechanism helps to keep the features that are learned from the contraction phase and use them for the reconstruction process. A 1 × 1 convolutional layer is used at the final layer of the network to map the final 64 feature vector to the targeted number of segmentation classes. In our case, the segmentation classes comprise two types: one is the background and the other is ICOS-positive cell. A total of 23 convolutional layers are used in the U-Net model.

**Figure 4:**
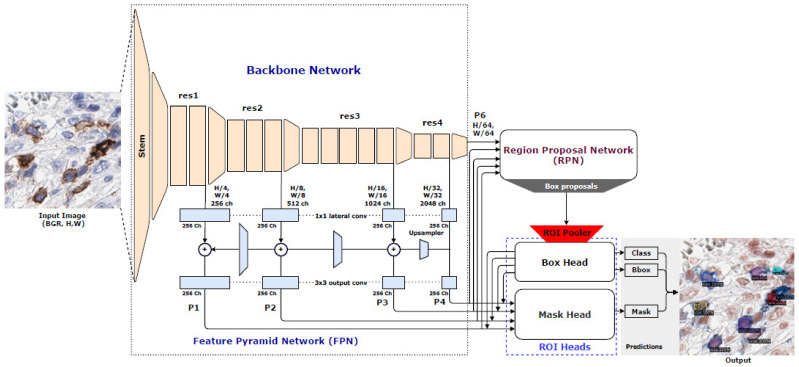
The architecture of Detectron2 has been modified from [27].

In the instance segmentation approach, Detectron2 [27] is a recent open-source instance segmentation system from Facebook AI Research. In our study, we use Faster R-CNN with Feature Pyramid Network (Base-RCNN-FPN) [28] for the bounding box detector and extend it to the Mask R-CNN [29] also for generating the segmentation mask in Detectron2. Therefore, it is a two-stage network that has three main blocks, namely, Backbone Network, Region Proposal Network (RPN), and ROI head, shown in Figure 4. To extract feature maps from the input image, we use the ResNet [30] architecture with FPN [28] as a Backbone Network. The ResNet model consists of a stem block and four bottleneck blocks. The stem block is used, having 77 convolution layers with the stride of 2. Afterward, a max-pooling layer with the stride of 2 is also used to down-sample the input image twice. The output feature map of the stem block is 64 × *H*/4 × *W*/4, where *H* and *W* represent the height and width of the input image. The four bottleneck blocks are used from the original ResNet architecture proposed in [30]. The FPN is composed of the four output features maps from the ResNet bottleneck blocks (*res1*, *res2*, *res3*, and *res4*), lateral, and output convolution layers. Each lateral convolution layer is used (1x1 convolution layer). It takes the output features from the bottleneck blocks with different channel numbers (256, 512, 1024, and 2048) and returns them to 256 channel feature maps. A forward process of the FPN begins from the *res4* output (see Figure 4); afterward, a 3 × 3 output convolution layer is used without changing the channel numbers. The resulted feature map list is *P4*. The output of *res4* is fed into the upsampler and added with the *res3* output by using lateral convolution. The resulting feature map is also fed to the output convolution and listed as *P3*. The process above is repeated two times more and the resulted feature maps are listed as *P2* and *P1*. The final *P5* output feature map is just a down-sample of the *res4* output by using a max-pooling layer with the stride of 2. The ROI head block is composed of two different heads: box head, and mask head, respectively. The box proposals are fed into the box head using the ROI pooling process. The final outputs of the box head are the class and the bounding box prediction scores. On the other hand, the four output features maps from FPN are used to feed into the mask head with the final outputs of the box head. The resulted prediction is to map the segmentation mask of the output object (e.g., ICOS cell). The final output image of Detectron2 contains three prediction maps corresponding to the class (object-level classification), bounding box (localization), and segmented mask (pixel-level classification) of the object.

### 2.5. Model Training

We train U-Net and Detectron2 individually on the PyTorch framework [31]. Initially, we use pre-trained weights from ResNet50 and ResNet101 [30] to train both models. We use the Stochastic Gradient Descent (SGD) [32] and Adam [33] optimizers with a dynamic learning rate of 0.002 reducing, based on the validation metric improvement. The Nesterov momentum of 0.9 and weight decay of 0.00003 is also used to accelerate the gradient descent. The Binary Cross-Entropy (BCE) and BCE with *L*1 norm loss function are used to train the U-Net and Detectron2 individually with the three different batch sizes, 2, 4, and 8 to find the best combinations of model hyper-parameters. Afterwards, we use different recent state-of-the-art pre-trained deep learning models (e.g., EfficientNetB7 [34], DenseNet161 [35], InceptionResNetV2 [36], SENetResNext101 [37], MobileNetV2 [38], and VGG19 [39]) as a backbone to train the U-Net with the Adam optimizer and the batch size of 4 and 8 to find the best pre-trained model. Note that when we change the backbone of U-Net, the encoder–decoder (see Figure 3) input and output feature maps are also changed with the corresponding pre-trained model configurations. Moreover, we also use different combinations of loss functions (e.g., Dice Coefficient (Dice), Intersection over Union (IoU), Focal, and Lovasz [40]) along with BCE for understanding the effect of the loss functions in deep learning models. Finally, the final U-Net is trained by three different train splits data sets, 100, 200, and 300, to investigate the data set size importance. To increase the size of the training data set, we augment the datasets by flipping the images horizontally and vertically, applying elastic transform, random and shift scale rotation with different values on the original input RGB images. Note that all the experiments were carried on two NVIDIA RTX 2080Ti GPU with 11GB memory, taking about 4–6 h to train 100 epochs for every individual model.

### 2.6. Post-Processing

Post-processing represents a major step in our workflow, it has an important effect on the performance of the model, particularly at the object level. To refine our final cell detection results, we start post-processing by applying distance transform on the segmented image; for each pixel, the distance transform assigns a number that represents the distance between that pixel and the nearest non-zero pixel. A common practice is then to apply the watershed algorithm; however, this often leads to an over-segmented image. The reason is each local minimum, no matter how small, becomes a catchment basin. To avoid over-segmentation, we first perform minima imposition to filter out tiny local minima and then modify the distance transform to have the minima at the desired locations only, before finalizing with the watershed. Minima imposition requires tuning the *H* parameter that controls the minimum depth value we should consider before applying the watershed. We adjust the *H* parameter through the extended minima transform, and through multiple experiments, we deduce the optimal value that provides the best precision and recall metrics.

### 2.7. Model Evaluation

In order to evaluate our models, we follow a multilevel validation strategy for the algorithms. This will allow us to build up and show algorithm performance as we go from the ‘raw’ network to the final output.

#### 2.7.1. Pixel-Level Validation

The U-Net and Detectron2 network provides a pixel-based output of the class probabilities of each pixel in the validation patches. This is converted into a segmentation map, typically using a threshold value. We use a threshold value of 0.5 to generate the final segmentation map. From this output we can obtain standard metrics based on the confusion matrix. We also plot a receiver operating characteristic (ROC) curve and calculate its area under the curve (AUC). The pixel performance metrics, accuracy (ACC), sensitivity (SEN) and specificity (SPE) are also calculated to evaluate models’ performances on pixel-level. Let the true positive (TP) rates be correctly classified pixels and the false positive (FP) rates be incorrectly classified pixels as the annotated ground-truth, whereas the true negative (TN) rates are correctly classified pixels and the false negative (FN) rates are incorrectly classified pixels as not the annotated ground-truth. The mathematical definitions of ACC, SEN, and SPE are presented as the following:

ACC = (TP + TN)/(TP + TN + FP + FN),(1)

SEN = TP/(TP + FN),(2)

SPE = TN/(TN + FP),(3)

#### 2.7.2. Object-Level Validation

For object-level validation, we post-process the probability map to identify the individual nucleus instances. This also involves thresholding, so we use this to plot the ROC for the model and use this to pick an optimal threshold for the confusion matrix-based metrics to calculate the Dice coefficient (Dice), aggregated Jaccard Index (AJI), precision and recall. For further analysis, we calculate the Dice (F1) score for each validation patch and plot this as a boxplot with overlaid scatter points in order to illustrate the variation and identify outliers to evaluate the models’ performance at the object level. We also rely on object-level metrics in order to compare deep learning based cell detection to ground-truth, which is represented by pathologist annotations. The mathematical definitions of Dice and AJI are presented as the following:

Dice = 2.TP/(2.TP + FP + FN),(4)

(5) $$\mathrm{AJI}\;=\frac{\sum_{i=1}^{N}\left|GT_{i}\;\cap \;PD_{j}^{*}\;\left(i\right)\right|}{\sum_{i=1}^{N}\left|GT_{i}\;\cup \;PD_{j}^{*}\;\left(i\right)\right|+\sum_{k\in I\;}\left|PD_{k}\right|}$$

Here, *GT_i_* is the *i*^th^ annotated ground-truth of nuclei pixels, *PD_k_ is* the predicted nuclei segmentation mask, PD*_j_(i) is the connected component from the predicted mask, and *I* is the list of indices of pixels that are not belong to the annotated ground-truth. The mathematical definitions of precision and recall are presented as the following:

Precision = TP/(TP + FP),(6)

Recall = TP/(TP + FN)(7)

## 3. Results

### 3.1. Comparative Analysis

The use of an AI algorithm to identify and quantify biomarker expression in cells/tissue must achieve high levels of accuracy on data that have not been used to train and develop the algorithm. Hence, we carry out a comprehensive evaluation of how deep learning models learn complex tissue features from the ICOS IHC tissue tiles and automatically identify the nuclear expression of IHC biomarkers ICOS. We carried out six different experiments to find the most robust deep learning model on the unseen test data set (all experimental details are presented in Table 1, Table 2, Table 3, Table 4, Table A1 and Table A2.

**Figure 5:**
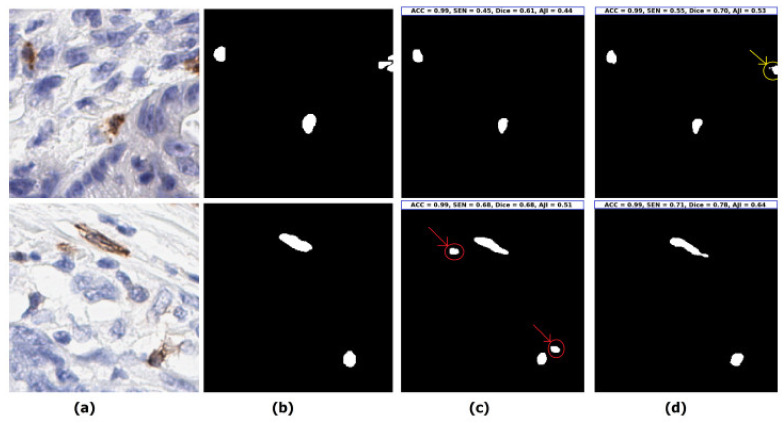
Visualization of two segmentation examples by the best U-Net and Detectron2 model from Table 1 for understanding the details of the model performances. (**a**) Original test images (randomly selected from the unseen test set), (**b**) ground-truth annotated by pathologist (binary regions corresponding to the original images), (**c**) predicted image by the Detectron2 model, individual image prediction with ACC, SEN, Dice, and AJI scores are presented on the top of the image (inside the blue box), the red arrow indicates the false positive and (**d**) predicted image by the U-Net model, individual image prediction with ACC, SEN, Dice, and AJI scores are presented on the top of the image (inside the blue box), yellow arrow indicates the model can segment the boundary connected cell.

Initially, we perform two-phase experiments of U-Net (semantic segmentation) and Detectron2 (instance segmentation) model training and testing with different training batches, optimizers, and loss functions to find the best approach (semantic segmentation vs. instance segmentation). The details are shown in Table 1. To evaluate the models, we use ACC, SEN and SPE for the pixel-level, and the Dice, AJI, precision and recall for the object-level validation metrics (details are explained in the model evaluation subsection in the Materials and Methods section). The U-Net model achieves the highest performance of 98.93%, 68.84%, and 53.92% in terms of ACC, Dice, and AJI using the backbone of ResNet101 with Adam optimizer, loss function of BCE, and the batch size of 8 on our unseen test dataset. On the other hand, Detectron2 yields the best performance of 99.63% of SPE, only using the backbone of ResNet50 with SGD optimizer, BCE+L1, and the batch size of 4. Moreover, the Adam and BCE loss yields a better performance than SGD and BCE with L1 loss. Figure 5 shows the segmentation performance between the Detectron2 and U-Net model. The upper row of the Figure 5 shows that the prediction of the Detectron2 model (3rd image) is not able to segment the boundary connected cells, whereas the U-Net model (4th image) can segment them. Moreover, the lower row shows that the Detectron2 model shows false positive results (more details are presented in Supplementary Materials Figure S1 and Figure S2). To summarize the first experiment, we show that the U-Net model (semantic segmentation approach) yields the best performance, compared to the Detectron2 model (instance segmentation approach) because the instance segmentation models are more precise on the global object identification (e.g., persons, vehicles, etc.), whereas semantic segmentation models consider the local level information more specifically. The U-Net style models, therefore, are more accurate in any cell segmentation task. 

Secondly, we then perform two sets of experiments of the U-Net model (see Table A1 (in Appendix A) and Table 2) with seven different state-of-the-art pretrained backbones (ResNet101, EfficientNetB7, DenseNet161, InceptionResNetV2, SENetResNext101, MobileNetV2, and VGG19), keeping the same optimizer Adam and loss function BCE, with the change of batch sizes of 4 and 8.

**Figure 6:**
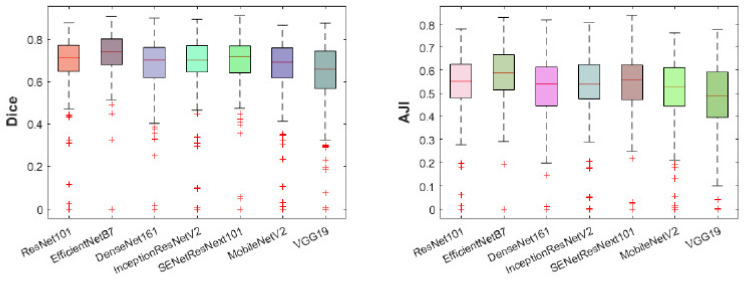
The box plots of Dice (**left**) and AJI (**right**) scores for all the test data sets. Different boxes with colours represent the different backbones of the U-Net model. The median value of every box is the red line inside the box; the outliers are labelled as the red (+) symbol.

**Figure 7:**
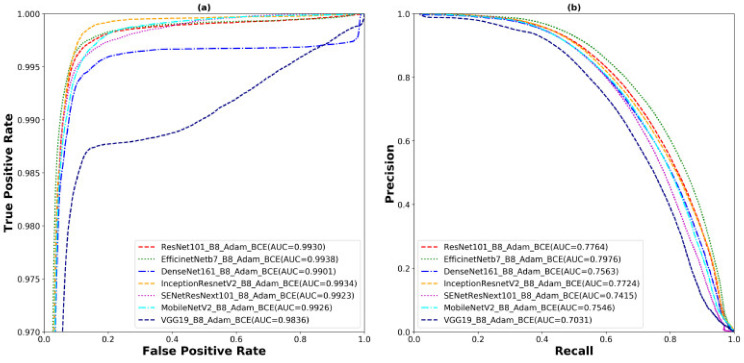
The (**a**) ROC and (**b**) PR carve for the experiment of the U-Net model with different backbones, the Adam optimizer, BCE loss function, and the batch size of 8.

**Figure A1:**
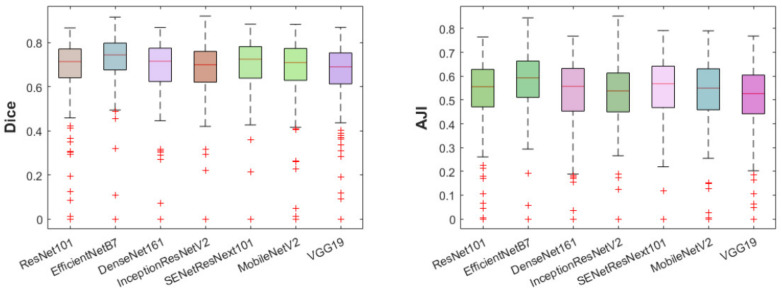
The box plots of Dice and AJI scores for all the test data sets. Different boxes with colours represent the different backbones of the U-Net model with the batch size of 4. The median value of every box is the red line inside the box; the outliers are labelled as the red (+) symbol.

The outcomes of these two experiments provides insights into the selection of the best pretrained backbone and batch size. U-Net with EfficientNetB7 backbone performs better than the other backbones using Adam, BCE, and batch sizes of 4 and 8 in both scenarios. It achieves 98.98%, 72.41%, 72.04%, and 57.50% in terms of ACC, SEN, Dice, and AJI, respectively, using a batch size of 4. On the other hand, it yields 98.99%, 73.92%, 72.44%, and 57.83% in terms of ACC, SEN, Dice, and AJI, respectively, using a batch size of 8. Increasing the batch size also improves the model performance of 0.01%, 1.51%, 0.4%, and 0.33% in terms of ACC, SEN, Dice, and AJI, respectively. However, the changing of the backbone from ResNet101 to EfficientNetB7 also improves the model performance of 3.6% and 3.91% of Dice and AJI, respectively. To conclude, the selection of the backbone and batch size is an important part of designing a robust deep learning model. The correct choice enhances the model performance. The best performance of the EfficientNetB7 backbone with a batch size of 8 is shown in Table 2. Detailed outliers of the Dice and AJI are illustrated in Figure 6, which shows that the EfficientNetB7 has the highest mean of Dice and AJI scores and the least standard deviation with some outliers. However, the rest of the models (ResNet101, DenseNet161, InceptionResNetV2, SENetResNext101, MobileNetV2, and VGG19) represent many outliers with a high standard deviation and low mean, compared to EfficientNetB7. The ROC and precision vs. recall (PR) curves are presented in Figure 7. The highest AUC of ROC is 99.38% and PR has 79.76% yields by the EfficientNetB7. Moreover, box plots and the ROC and PR curves for the batch size of 4 are presented in Appendix A Figure A1, and Supplementary Materials Figure S3, respectively.

**Figure 8:**
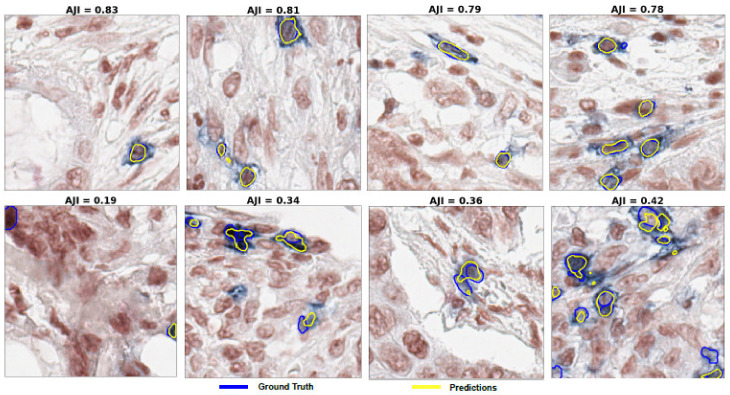
Examples of some segmentation results of the optimized U-Net model using the test data set. (**Upper row**): the best performance of the model. (**Lower row**): the poor performance of the model. Note that, the blue and yellow colours represent the annotated ground-truth and the best model predictions, respectively.

**Figure A2:**
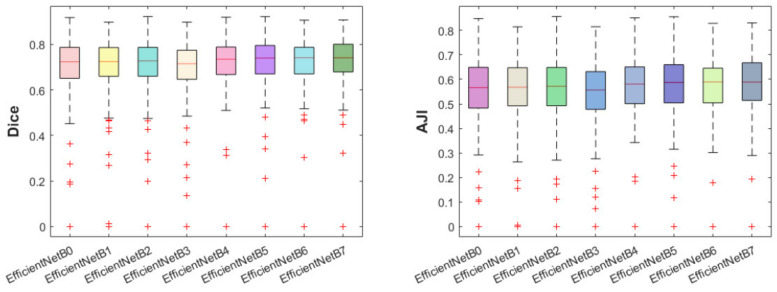
The box plots of Dice and AJI scores for all the test data sets. Different boxes with colours represent the different versions of the EfficientNet backbone. The median value of every box is the red line inside the box; the outliers are labelled as the red (+) symbol.

Thirdly, we then run further experiments to understand the effect of the model size (see Table A2 (in Appendix A)). We select the same experimental configurations as that previously used (Table 2) but change the EfficientNet backbone from B0 (lightweight) to B7 (heavyweight). The results show that EfficientNet, B0 (lightweight) achieved an AJI of 55.18%, whereas the B7 (heavyweight) yielded an AJI of 57.83%, meaning that it improved 2.73% of an AJI using a heavyweight model. Detailed outliers of the Dice and AJI are illustrated in Appendix A Figure A2, which shows that the EfficientNetB7 has the highest mean of Dice and AJI scores and the least standard deviation with some outliers. Fourthly, we show the effect of the loss function on the optimized U-Net model from previous experiments (Table 2). The detailed experimental outcomes are presented in Table 3. The model improved the performance of Dice and AJI by 0.99% and 1.15%, respectively, using both BCE and IoU loss, compared with the BCE loss. The BCE and Dice loss also improved the SEN by 8.96%. The BCE and Focal loss yielded the ACC and SPE of 0.02% and 0.44% respectively, higher than the BCE and IoU loss. To summarize, we obtained our best optimized U-Net model with the backbone of EfficientNetB7, Adam, BCE with IoU loss and the batch size of 8. Some segmentation examples of our best model are shown in Figure 8. The results show that the performance for the segmentation was better when the cells were well separated. In turn, the close distance and boundary-connected cell segmentation performance were comparatively poor.

We perform post-processing for separating every individual cell accurately to calculate the density of the cells, which leads us to develop a robust density estimation and survival analysis system (see in Correlation and Survival Analysis subsections). 

**Figure 9:**
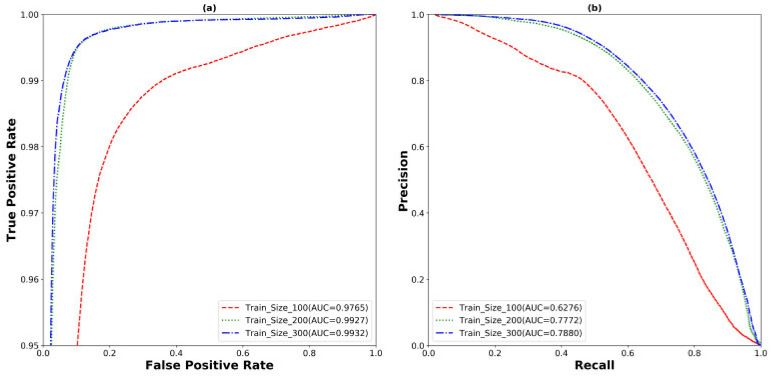
The (**a**) ROC and (**b**) PR curve for the experiment of different training sizes.

Finally, we show the effect of the trained data set size by using our optimized U-Net model. For this experiment, we split the training data set into three different sizes, 100, 200, and 300. Every split is tested with the same test data set and presented in Table 4. The SEN, Dice, and AJI scored 51.28%, 55.60%, and 40.73% with the 100, 69.84%, 70.29%, and 55.45% with the 200 and 71.85%, 71.44%, and 56.66% with the 300 training size, respectively. The performance improved dramatically from the training size 100 to 200 with the 12.56%, 14.69%, and 14.72% increment of SEN, Dice, and AJI, respectively. There was also an improvement of 2.01%, 1.15%, and 1.21% of SEN, Dice, and AJI from training size 200 to 300. However, the experiments show that the increase in the training data set size was also important in improving the model performance. The effect of the training size experiments is illustrated by the ROC and PR curves in Figure 9.

The highest AUC of ROC is 99.32% and PR is 78.80% yielded by the training size 300. However, we have still the challenge of segmenting the separate cells when they are very close to each other. Therefore, we use another post-processing step (details are explained in the next subsection) to separate these connected cells from the model predictions.

### 3.2. Object-Level Performance Evaluation after Post-Processing

**Figure 10:**
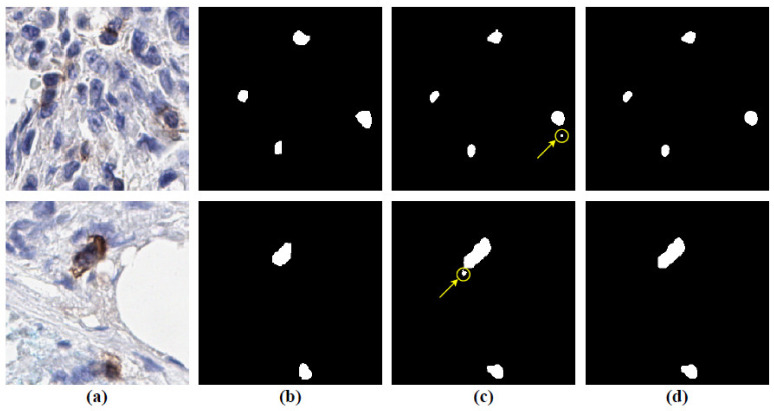
Visualization of the effect of post-processing method using two segmentation examples. (**a**) Original test images (randomly selected from the unseen test set), (**b**) annotated ground-truth by pathologist (binary regions corresponding to the original images), (**c**) predicted images by the model before applying the post processing method; yellow arrows are indicating the false positives are detection and (**d**) predicted images after applying the post-processing method; the detected false positives are removed.

We evaluate our model performance at the object level by estimating precision and recall. Recall measures the ratio of objects, in our case positive cells, that are correctly detected to the total number of objects. Precision describes how good the model is in evaluating the performance of the best model at the object level. The details of the post-processing method are explained in the post-processing subsection under the Method section. As explained in post processing, the precision and recall are evaluated using different values of extended regional minima index H. We find that the optimal value for ICOS cell detection is H = 6. For comparison, we use precision and recall to evaluate the performance of the method used in [21] for cell segmentation. The ground truth images used for evaluation are annotated and reviewed by expert pathologists. Precision varies from 32.61% to 34.82%, whereas, when applying the selected nuclear segmentation deep learning model, it is between 67.23% and 83.33% with the best model. In the same way, recall does not exceed 3.82% for the nuclear segmentation tool but reaches 66.02% with the best model. Table 5 provides examples for both methods, with varying thresholds. Figure 10 illustrates examples of the detection results, along with their corresponding ground-truths, which are annotations from the pathologist. We can clearly see that deep learning, followed by post-processing workflow described above, refines the noisy regions from the model predictions.

### 3.3. Correlation Analysis—Clinical Relevance

**Figure 11:**
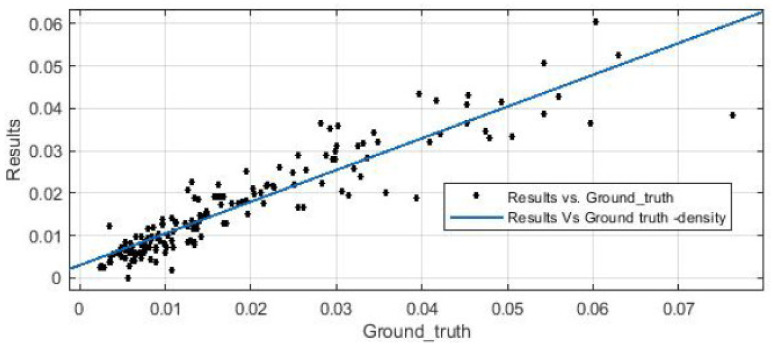
Density estimation for post-processing results vs. ground-truth annotations.

In this section, we evaluate the Pearson correlation coefficient (R) in order to measure their linear dependence, and find R = 0.9244, which denotes a high dependency, and consequently, that our ICOS density estimation is highly accurate, with respect to the annotations provided by the pathologist. Figure 11 illustrates the predicted density from our workflow against the ground-truth density from pathologist annotations.

### 3.4. Survival Analysis

**Figure 12:**
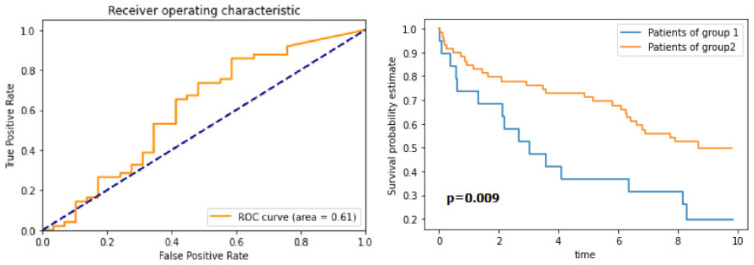
The ROC for the optimal cut-off value of the ICOS density (**left-image**), and the Kaplan–Meier curves for the survival analysis (**right-image**) (group 1 above threshold and group 2 below the threshold).

Given the high concordance between human generated annotations and our final results, we look to validate our workflow with a survival analysis of subset of 97 patients (equivalent to a single TMA array) from the cohort used by [21]. We use the detected cells to estimate the density of positive cells per mm^2^ within every patient TMA core. Using these scores, and following the approach taken in [21], we then perform time-dependent ROC curve analysis, using the censored overall survival data. The ROC curve, shown in Figure 12, allows the optimal cut-off value for the ICOS density to be estimated. This threshold value is used to separate the patients into two groups for overall survival analysis. Using these groups as defined at the optimal threshold, we can clearly see that the scores based on the deep learning detections could be used to stratify the cases into two groups which, when analysed using Kaplan–Meier curves, show a marked difference in survival (Figure 12). To test for statistical significance of the difference between the survival curves, we run the log-rank test, based on 5-year survival. The calculated p-value of the log-rank test is *p* = 0.009, which indicates statistical significance and compares well with [21]. Given this statistically significant result, we conclude that the workflow presented in this paper allows for the development of deep learning-based IHC scoring algorithms, which have potential value in determining biomarker prognostication, following validation in future studies.

## 4. Discussion and Conclusions

In this study, we have presented a complete workflow for immune-checkpoint ICOS protein detection in CRC. After performing a set of experiments to find the most appropriate approach of deep learning architectures for ICOS cell detection, we documented the effect of the different pre-trained backbones, batch size, loss functions, and train data set sizes for training a deep learning model. Based on the performance metrics at both the pixel and object levels, pixel segmentation proved to be superior; the U-Net architecture trained and tested with the EfficientNetB7 backbone, Adam optimizer, BCE loss, and the batch size of 8 provided the best results. Furthermore, we compared our deep learning model results to the ground-truth data, that is, a set of annotations provided by the pathologist, and concluded that, after post-processing, ICOS positive cell detection provided results very close to those of the pathologist. We concluded with the density estimation of ICOS-positive cells and confirmed the high accuracy of our results, by measuring the correlation coefficient. Given the strong concordance between the hand-generated annotations and the deep learning model output, we can confidently use our results on pathological images associated with robust clinical metadata. Our current model using this workflow was shown to predict overall survival for these stage II/III CRC patients. The use of survival analysis based on our model extends the usual technical validation using segmentation and correlation metrics and provides a useful example of how deep-learning-based models may be used to develop prognostic and predictive models through robust development processes.

## Figures and Tables

**Table 1:** A performance comparison between the U-Net and Detectron2 on the test data set, using different combinations of backbone, batch size, optimizer and loss functions (bold represent the best performance).

Model Name	Backbone	Batch Size	Optimizer	Loss Function	Metrics				
					Accuracy	Sensitivity	Specificity	Dice	AJI
U-Net	ResNet50	2	SGD	BCE	0.97135	**0.81653**	0.97349	0.51159	0.35148
		4			0.97729	0.66402	0.98229	0.5015	0.34194
		8			0.98119	0.19418	0.99565	0.25376	0.14849
		2	Adam	BCE	0.98922	0.65317	0.99571	0.67643	0.5301
		4			0.98933	0.65703	0.99585	0.67773	0.53082
		8			0.98904	0.65452	0.99551	0.66949	0.52215
	ResNet101	2	SGD	BCE	0.97202	0.80081	0.97434	0.51812	0.35626
		4			0.97635	0.71966	0.98038	0.52575	0.36193
		8			0.98066	0.20058	0.99493	0.25703	0.15036
		2	Adam	BCE	0.98902	0.65415	0.99538	0.67106	0.52394
		4			0.98903	0.66182	0.99542	0.67364	0.52681
		8			**0.98939**	0.67254	0.99584	**0.68844**	**0.53922**
Detectron2	ResNet50	2	SGD	BCE+L1	0.98795	0.63321	0.99509	0.6571	0.50428
		4			0.98823	0.58092	**0.99632**	0.63354	0.48617
		8			0.98816	0.57355	0.99629	0.62887	0.48037
		2	Adam	BCE+L1	0.98811	0.63597	0.99514	0.65672	0.50619
		4			0.98792	0.57015	0.99616	0.61928	0.47275
		8			0.98823	0.58092	0.99632	0.63354	0.48617
	ResNet101	2	SGD	BCE+L1	0.9881	0.62078	0.99563	0.65472	0.50358
		4			0.98778	0.5791	0.99607	0.62088	0.47237
		8			0.98788	0.58846	0.99609	0.63493	0.48353
		2	Adam	BCE+L1	0.98828	0.62597	0.99563	0.65985	0.50696
		4			0.98817	0.59231	0.9963	0.63697	0.48881
		8			0.98815	0.59255	0.99622	0.63644	0.48773

**Table 2:** Comparative results of U-Net with the different backbones and the batch size of 8 (bold represent the best performance).

Model Name	Backbone	Batch Size	Optimizer	Loss Function	Metrics				
					Accuracy	Sensitivity	Specificity	Dice	AJI
U-Net	ResNet101	8	Adam	BCE	0.98939	0.67254	0.99584	0.68844	0.53922
	EfficientNetB7				**0.98992**	0.7392	0.99526	**0.72448**	**0.57832**
	DenseNet161				0.98881	0.66545	0.99521	0.66838	0.51961
	InceptionResNetV2				0.98918	0.66615	0.99553	0.67742	0.52953
	SENetResNext101				0.98812	****0.74222****	0.99331	0.67823	0.53138
	MobileNetV2				0.98891	0.63465	**0.99589**	0.65913	0.50924
	VGG19				0.98778	0.5761	0.99568	0.61238	0.46402

**Table 3:** Comparative results of U-Net with different loss functions (bold represents the best performance).

Model Name	Backbone	Batch Size	Optimizer	Loss Function	Metrics				
					Accuracy	Sensitivity	Specificity	Dice	AJI
U-Net	EfficientNetB7	8	Adam	BCE+Dice	0.98894	**0.82885**	0.99238	0.72865	0.58277
				BCE+IoU	0.98931	0.81816	0.99305	**0.73447**	**0.58986**
				BCE+DICE+IoU	0.98893	0.81521	0.99249	0.72694	0.58024
				BCE+Focal	**0.98953**	0.60196	**0.99745**	0.66682	0.51891
				BCE+Lovasz	0.98874	0.81792	0.99226	0.72196	0.57535
				BCE+Dice+IoU+Focal	0.98916	0.81891	0.99286	0.7301	0.5845

**Table 4:** A performance comparison of U-Net in different train sizes (bold represents the best performance).

Model Name	Backbone	Batch Size	Optimizer	Loss Function	Train Size	Metrics				
						Accuracy	Sensitivity	Specificity	Dice	AJI
U-Net	EfficientNetB7	8	Adam	BCE	100	0.98627	0.5128	**0.99564**	0.55607	0.40732
					200	0.98942	0.69842	0.99547	0.70291	0.5545
					300	**0.98968**	**0.71852**	0.99534	**0.71443**	**0.56669**

**Table 5:** Object-level performance metrices after applying the post-processing method.

Threshold	Precision (%)	Recall (%)
0.30	83.33	66.02
0.35	80.71	63.95
0.40	76.22	60.39
0.45	72.1	57.12
0.50	67.23	53.26

## Data Availability

The samples used are part of the Epi700 colon cancer cohort, and were received from the Northern Ireland Biobank. Data availability is subject to an application to the Northern Ireland Biobank.

## Supplementary Materials

The following are available online at https://www.mdpi.com/article/10.3390/cancers13153825/s1. Figure S1: The ROC space plot for the experiment 1 (comparison between the U-Net and Detectron2 performance on the test dataset using different combinations of backbone, batch size, optimizer and loss functions). The point that is closet to (0,1) and found U-Net with the backbone of ResNet101, Adam, BCE and batch size of 8, which is considered as an “optimal” performance., Figure S2: Comparison of segmentation results of the best U-Net and Detectron2 model from the experiment 1. (a) Original test image (randomly selected from the unseen test set), (b) annotated ground-truth (binary regions corresponding to the original images), (c) predicted image by the Detectron2 model, individual image prediction with ACC, SEN, Dice, and AJI scores are presented on the top of the image, (d) predicted image by the U-Net model, individual image prediction with ACC, SEN, Dice, and AJI scores are presented on the top of the image. Figure S3: The (a) ROC and (b) PR carves for the experiment of U-Net model with different backbones, the Adam optimizer, BCE loss function and the batch size of 4. Source Code S1.1: The source code for U-Net model. Source Code S1.2: The source code for Detectron2 model.

## Abbreviations

ICOS	Immune-checkpoint Inducible T-cell COStimulator
AI	Artificial Intelligence
CRC	Colorectal Cancer
IHC	Immunohistochemistry
TMA	Tissue Microarrays
ReLU	Rectified Linear Unit
CNN	Convolutional Neural Network
R-CNN	Region-based Convolutional Neural Network
ROI	Region of interest (ROI)
RPN	Region Proposal Network
FPN	Feature Pyramid Network
SGD	Stochastic Gradient Descent
BCE	Binary Cross-Entropy
Dice	Dice Coefficient
IoU	Intersection over Union
ROC	Receiver Operating Characteristic
AUC	Area Under the Curve
ACC	Accuracy
SEN	Sensitivity
SPE	Specificity
AJI	Aggregated Jaccard Index
GPU	Graphics Processing Unit
GB	Gigabytes
RGB	Red, Blue and Green
DL	Deep Learning

## Appendix A. Experiments

In this experiment, the U-Net model with seven different state-of-the-art pre-trained back bones is used with Adam, BCE, and the batch size of 4. The details of the outliers of Dice and AJI are shows in Figure A1.

**Table A1:** Comparative results of U-Net with the different backbones and the batch size of 4 (bold represent the best performance).

Model Name	Backbone	Batch Size	Optimizer	Loss Function	Metrics				
					Accuracy	Sensitivity	Specificity	Dice	AJI
U-Net	ResNet101	4	Adam	BCE	0.98903	0.66182	0.99542	0.67364	0.52681
	EfficientNetB7				**0.98989**	**0.72419**	0.99548	**0.7204**	**0.57509**
	DenseNet161				0.98935	0.65444	0.99592	0.67743	0.53043
	InceptionResNetV2				0.98738	0.72407	0.99203	0.66439	0.5154
	SENetResNext101				0.98938	0.68371	0.99579	0.69138	0.54407
	MobileNetV2				0.98926	0.65939	0.99563	0.67371	0.52711
	VGG19				0.98892	0.61574	**0.99608**	0.65206	0.50478

In this experiment, the U-Net with the different depth size of EfficientNet backbones are used with Adam, BCE, and the batch size of 8 to understand the importance of the network depths. The results show in Table A2 that the deeper network can help to improve the U-Net segmentation performance. Hence, the details of the outliers of Dice and AJI are illustrated in Figure A2.

**Table A2:** A performance comparison of U-Net with the different backbone versions of EfficientNet (bold represent the best performance).

Model Name	Backbone	Batch Size	Optimizer	Loss Function	Metrics				
					Accuracy	Sensitivity	Specificity	Dice	AJI
U-Net	EfficientNetB0	8	Adam	BCE	0.98945	0.70358	0.99528	0.69887	0.5518
	EfficientNetB1				0.98948	0.71624	0.9952	0.7045	0.55721
	EfficientNetB2				0.98962	0.70494	0.99539	0.70469	0.5579
	EfficientNetB3				0.98953	0.68548	**0.99597**	0.69697	0.54769
	EfficientNetB4				0.98988	0.72678	0.99543	0.71955	0.57232
	EfficientNetB5				0.98987	0.73887	0.9951	0.72172	0.5768
	EfficientNetB6				0.98978	0.73786	0.99528	0.72114	0.57353
	EfficientNetB7				**0.98992**	**0.7392**	0.99526	**0.72448**	**0.5783**
