# Supplementary material for: A Means of Assessing Deep Learning-Based Detection of ICOS Protein Expression in Colon Cancer
Source: Cancers (Basel). 2021 Jul 29;13(15):3825. doi: 10.3390/cancers13153825 (PMC8345140; doi:10.3390/cancers13153825)
Supplement: Supplementary file 1 [file cancers-13-03825-s001.zip › cancers-1288048-supplementary.pdf]

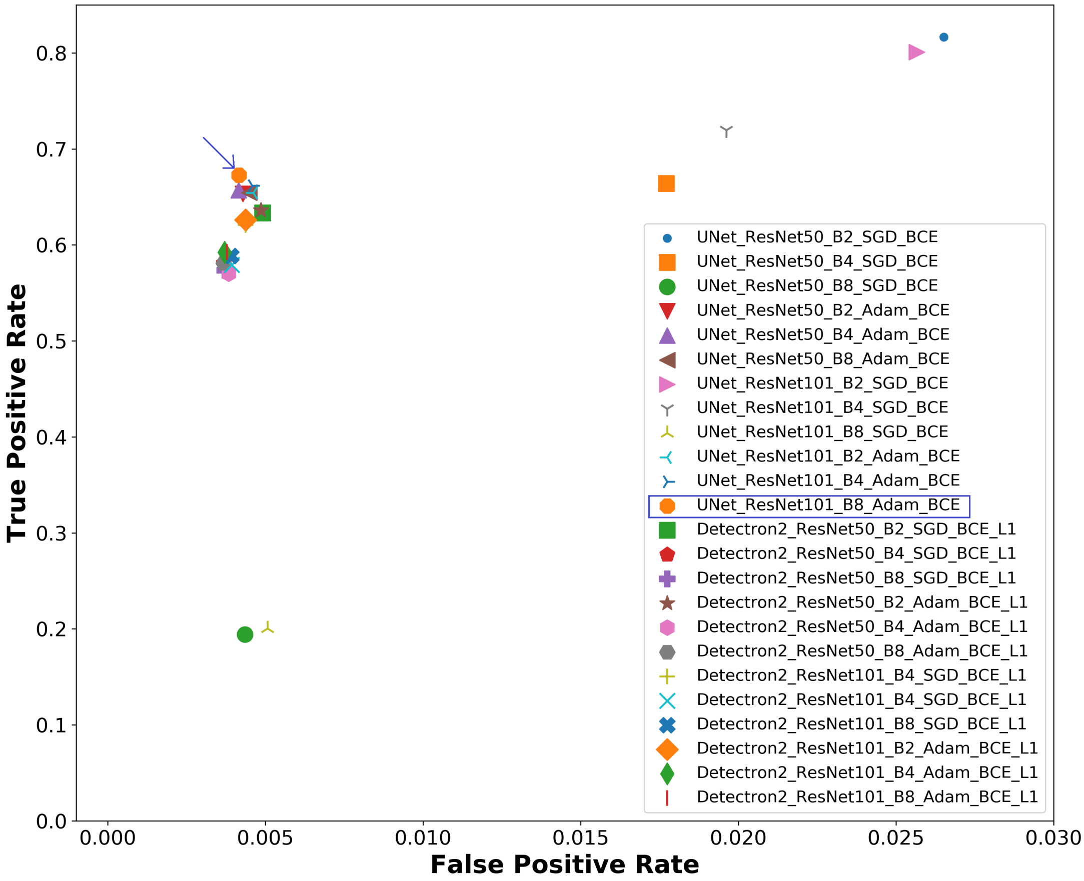

Figure S1: The ROC space plot for the experiment 1 (comparison between the U-Net and Detectron2 performance on the test dataset using different combinations of backbone, batch size, optimizer and loss functions). The point that is closet to (0,1) and found U-Net with the backbone of ResNet101, Adam, BCE and batch size of 8 which is considered as an “optimal” performances.

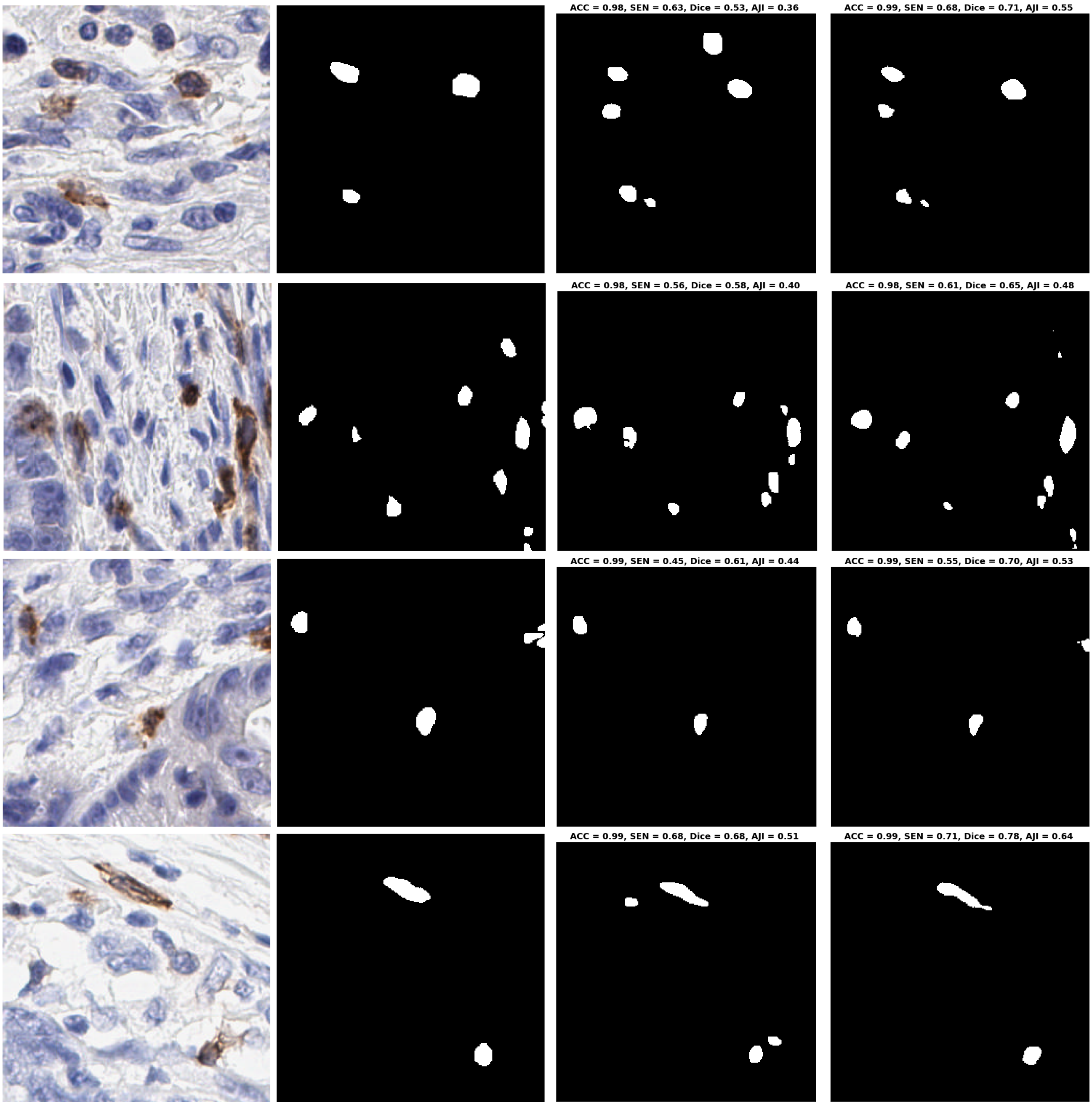

Figure S2: Comparison of segmentation results of the best U-Net and Detectron2 model from the experiment 1. (a) Original test image (randomly selected from the unseen test set), (b) Annotated ground truth (binary regions corresponding to the original images), (c) Predicted image by the Detectron2 model, individual image prediction with ACC, SEN, Dice, and AJI scores are presented on the top of the image, (d) Predicted image by the U-Net model, individual image prediction with ACC, SEN, Dice, and AJI scores are presented on the top of the image.

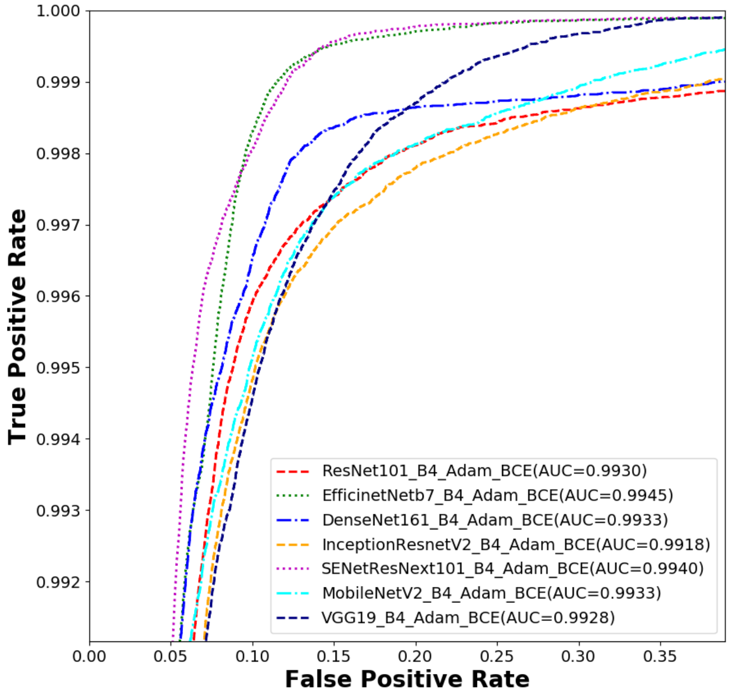

(a)

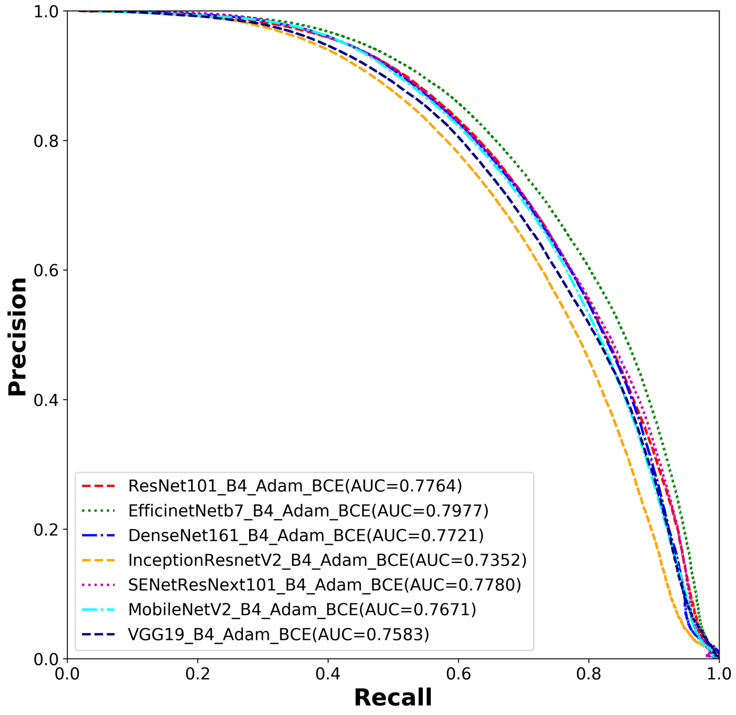

(b)

Figure S3: The ROC and PR carves for the experiment of U-Net model with different backbones, the Adam optimizer, BCE loss function and the batch size of 4.

1 Source Code

1.1 The source code for U-Net model:

In [ ]:

```
"""
This python file for traiing and test ICOS positive cell semantic segmentation by U-Net model.

@author: Md Mostafa Kamal Sarker (PhD)
Research Fellow
Tissue Hybridisation & Digital Pathology
Precision Medicine Centre of Excellence
Queen's UniversityBelfast,UK.
Email: M.Sarker@qub.ac.uk
@ Date: 10.01.2021

"""

# important library installation for train, validate and test the model U-Net model
# Catalyst
!pip install catalyst==20.12

# for data augmentations
!pip install albumentations==0.4.3

# for pretrained segmentation models for PyTorch
!pip install segmentation-models-pytorch==0.1.0

# for TTA
!pip install ttach==0.0.2

# for tensorboard
!pip install tensorflow
```

In [ ]:

```python
## import important libraries
from typing import Callable, List, Tuple
import os
import torch
import catalyst
from catalyst import utils
print(f"torch: {torch.__version__}, catalyst: {catalyst.__version__}")
# os.environ["CUDA_VISIBLE_DEVICES"] = "0"  # "" - CPU, "0" - 1 GPU, "0,1" - MultiGPU
SEED = 123
utils.set_global_seed(SEED)
utils.prepare_cudnn(deterministic=True)
```

In [ ]:

```
### Set the dataset paths for ICOS
from pathlib import Path
## define the root floder
ROOT = Path("../Data/")
## training dataset
train_image_path = ROOT / "train/images"
train_mask_path = ROOT / "train/mask"
## validation dataset is used for validation during model training
trainval_image_path = ROOT / "trainval/images"
trainval_mask_path = ROOT / "trainval/mask"
# test dataset keep unseen for the model dusring the training and vlidate.
## only use for the test
test_image_path = ROOT / "test/images"

### check the train images
print('***TRAIN***')
TRAIN_IMAGES = sorted(train_image_path.glob("*.png"))
print(len(TRAIN_IMAGES))
### check the masks
TRAIN_MASKS = sorted(train_mask_path.glob("*.png"))
print(len(TRAIN_MASKS))
### val
### check the images
print('***TRAINVAL***')
TRAINVAL_IMAGES = sorted(trainval_image_path.glob("*.png"))
print(len(TRAINVAL_IMAGES))
### check the masks
TRAINVAL_MASKS = sorted(trainval_mask_path.glob("*.png"))
print(len(TRAINVAL_MASKS))
```

In [ ]:

```python
import random
import matplotlib.pyplot as plt
import numpy as np
from skimage.io import imread as mask_imread
from catalyst import utils

## visualizations functions
def show_examples(name: str, image: np.ndarray, mask: np.ndarray):
    plt.figure(figsize=(10, 14))
    plt.subplot(1, 2, 1)
    plt.imshow(image)
    plt.title(f"Image: {name}")

    plt.subplot(1, 2, 2)
    plt.imshow(mask)
    plt.title(f"Mask: {name}")


def show(index: int, images: List[Path], masks: List[Path], transforms=None) -> None:
    image_path = images[index]
    name = image_path.name

    image = utils.imread(image_path)
    mask = mask_imread(masks[index])

    if transforms is not None:
        temp = transforms(image=image, mask=mask)
        image = temp["image"]
        mask = temp["mask"]

    show_examples(name, image, mask)

def show_random(images: List[Path], masks: List[Path], transforms=None) -> None:
    length = len(images)
    index = random.randint(0, length - 1)
    show(index, images, masks, transforms)
```

In [ ]:

```python
show_random(TRAIN_IMAGES, TRAIN_MASKS)
```

```
In [ ]: import albumentations as albu
from albumentations.pytorch import ToTensor
from typing import List
from torch.utils.data import Dataset

## define the dataloader for the model
class SegmentationDataset(Dataset):
    def __init__(
        self,
        images: List[Path],
        masks: List[Path] = None,
        transforms=None
    ) -> None:
        self.images = images
        self.masks = masks
        self.transforms = transforms

    def __len__(self) -> int:
        return len(self.images)

    def __getitem__(self, idx: int) -> dict:
        image_path = self.images[idx]
        image = utils.imread(image_path)

        result = {"image": image}

        if self.masks is not None:
            mask = mask_imread(self.masks[idx])
            result["mask"] = mask

        if self.transforms is not None:
            result = self.transforms(**result)

        result["filename"] = image_path.name

        return result

def pre_transforms(image_size=256):
    return [albu.Resize(image_size, image_size, p=1)]

def hard_transforms():
```

```
    result = [
      albu.RandomRotate90(),
        albu.VerticalFlip(),
        albu.HorizontalFlip(),
        albu.ElasticTransform(p=0.75),
        albu.ShiftScaleRotate(p=0.75)
    ]

    return result

def post_transforms():
    # we use ImageNet image normalization
    # and convert it to torch.Tensor
    return [albu.Normalize(), ToTensor()]

def compose(transforms_to_compose):
    # combine all augmentations into single pipeline
    result = albu.Compose([
      item for sublist in transforms_to_compose for item in sublist
    ])
    return result
```

In [ ]:

```
## apply transform and visualize
train_transforms = compose([hard_transforms(),post_transforms()])
valid_transforms = compose([pre_transforms(), post_transforms()])
show_transforms = compose([hard_transforms()])
show_random(TRAIN_IMAGES, TRAIN_MASKS, transforms=show_transforms)
```

In [ ]:
```
import collections
from sklearn.model_selection import train_test_split
from torch.utils.data import DataLoader

## define the batch size
batch_size: int = 8
## Define the number of workers
num_workers: int = 0

## dataloader
def get_loaders(
    train_images: List[Path],
    train_masks: List[Path],
    trainval_images: List[Path],
    trainval_masks: List[Path],
    batch_size: batch_size,
    num_workers: num_workers,
    train_transforms_fn = None,
    valid_transforms_fn = None,
) -> dict:

    train_indices = np.arange(len(train_images))
    trainval_indices = np.arange(len(trainval_images))

    np_train_images = np.array(train_images)
    np_train_masks = np.array(train_masks)
    np_trainval_images = np.array(trainval_images)
    np_trainval_masks = np.array(trainval_masks)

    # Creates our train dataset
    train_dataset = SegmentationDataset(
      images = np_train_images[train_indices].tolist(),
      masks = np_train_masks[train_indices].tolist(),
      transforms = train_transforms_fn
    )

    # Creates our valid dataset
    valid_dataset = SegmentationDataset(
      images = np_trainval_images[trainval_indices].tolist(),
      masks = np_trainval_masks[trainval_indices].tolist(),
      transforms = valid_transforms_fn
```

```
    )

    # Catalyst uses normal torch.data.DataLoader
    train_loader = DataLoader(
      train_dataset,
      batch_size=batch_size,
      shuffle=True,
      num_workers=num_workers,
      drop_last=True,
    )

    valid_loader = DataLoader(
      valid_dataset,
      batch_size=batch_size,
      shuffle=False,
      num_workers=num_workers,
      drop_last=True,
    )

    # And excpect to get an OrderedDict of loaders
    loaders = collections.OrderedDict()
    loaders["train"] = train_loader
    loaders["valid"] = valid_loader

    return loaders

loaders = get_loaders(
    train_images=TRAIN_IMAGES,
    train_masks=TRAIN_MASKS,
    trainval_images=TRAINVAL_IMAGES,
    trainval_masks=TRAINVAL_MASKS,
    train_transforms_fn=train_transforms,
    valid_transforms_fn=valid_transforms,
    num_workers=  num_workers,
    batch_size=batch_size
)
```

In [ ]:
```
import warnings
warnings.filterwarnings('ignore')
import segmentation_models_pytorch as smp
from torch import nn
from torch import optim
from catalyst.contrib.nn import RAdam, Lookahead
from catalyst.contrib.nn import  IoULoss
from catalyst.dl import SupervisedRunner
from catalyst.dl import DiceCallback, IouCallback, \
  CriterionCallback, MetricAggregationCallback, EarlyStoppingCallback
from catalyst.contrib.callbacks import DrawMasksCallback

### pretrained U-Net Model
# The U-Net Network with pre-trained efficientnet-b7 backbone (can change any other backbones)
model = smp.Unet(encoder_name="efficientnet-b7", classes=1)

# used BCE and IoU loss function (can change the loss functions)
criterion = {
    "bce": nn.BCEWithLogitsLoss(),
    "iou": IoULoss(),
}

learning_rate = 0.002
encoder_learning_rate = 0.0001

# Since we use a pre-trained encoder, we will reduce the learning rate on it.
layerwise_params = {"encoder*": dict(lr=encoder_learning_rate, weight_decay=0.00003)}

# This function removes weight_decay for biases and applies our layerwise_params
model_params = utils.process_model_params(model, layerwise_params=layerwise_params)

# optimizers
base_optimizer = optim.Adam(model.parameters(), lr=learning_rate, weight_decay=0.00003)
optimizer = Lookahead(base_optimizer)

scheduler = optim.lr_scheduler.ReduceLROnPlateau(optimizer, factor=0.1, patience=5)

## Epochs
num_epochs = 100
## log dir to save and load checkpoint
logdir = "./logs/U-Net_EfficientNet_efficientnetb7_B8_Adam_BCE_IoU"
```

```python
device = utils.get_device()
print(f"device: {device}")

# by default SupervisedRunner uses "features" and "targets",
# in our case we get "image" and "mask" keys in dataset __getitem__
runner = SupervisedRunner(device=device, input_key="image", input_target_key="mask")
callbacks = [
    # Each criterion is calculated separately.
    CriterionCallback(
        input_key="mask",
        prefix="loss_bce",
        criterion_key="bce"
    ),
    CriterionCallback(
        input_key="mask",
        prefix="loss_iou",
        criterion_key="iou"
    ),

    # And only then we aggregate everything into one loss.
    MetricAggregationCallback(
        prefix="loss",
        mode="weighted_sum", # can be "sum", "weighted_sum" or "mean"
        # because we want weighted sum, we need to add scale for each loss
        metrics={"loss_bce": 0.2, "loss_iou": 0.8},
    ),

    # metrics
    DiceCallback(input_key="mask"),
    IouCallback(input_key="mask"),
    # visualization
    DrawMasksCallback(output_key='logits',
                      input_image_key='image',
                      input_mask_key='mask',
                      summary_step=50
    ),
    ## early stop
    EarlyStoppingCallback(patience=5, metric="dice", minimize=False)
]
```

```python
## for training the model (for standalone test purpose please close this section)
runner.train(
    model=model,
    criterion=criterion,
    optimizer=optimizer,
    scheduler=scheduler,
    # our dataloaders
    loaders=loaders,
    # We can specify the callbacks list for the experiment;
    callbacks=callbacks,
    # path to save logs
    logdir=logdir,
    num_epochs=num_epochs,
    # save our best checkpoint by IoU metric
    main_metric="dice",
    # IoU needs to be maximized.
    minimize_metric=False,
    # prints train logs
    verbose=True,
)
```

In [ ]:

```
## perform test with the unseen test dataset.

TEST_IMAGES = sorted(test_image_path.glob("*.png"))

# create test dataset
test_dataset = SegmentationDataset(
    TEST_IMAGES,
    transforms=valid_transforms
)

## test data loader
infer_loader = DataLoader(
    test_dataset,
    batch_size=batch_size,
    shuffle=False,
    num_workers=num_workers
)

# this get predictions for the whole loader
predictions = np.vstack(list(map(
    lambda x: x["logits"].cpu().numpy(),
    runner.predict_loader(loader=infer_loader, model=model, resume=f"{logdir}/checkpoints/best.pth")
)))
```

In [ ]:

```
from PIL import Image
## save predict dir path
save_dir= f"{logdir}/test_results/"
threshold = 0.5  ## apply threshold

for i, (features, logits) in enumerate(zip(test_dataset, predictions)):
    filename =features['filename']
    print(filename)
    image = utils.tensor_to_ndimage(features["image"])
    mask_ = torch.from_numpy(logits[0]).sigmoid()
    mask = utils.detach(mask_ > threshold).astype("float")
    mask =mask*255
    mask = np.asarray(mask, dtype=np.int8)
    mask= Image.fromarray(mask).convert('RGB')
    if not os.path.exists(save_dir):
        os.makedirs(save_dir)
    # ## save binary image for calculating the validation scores and visualizations
    mask.save(save_dir+filename)

#     show_examples(name="", image=image, mask=mask)  # for
```

In [ ]:

1.2 The source code for Detectron2 model:

In [ ]:
```
"""
This python file for traiing and test ICOS positive cell instance segmentation by Detectron2 model.

@author: Md Mostafa Kamal Sarker (PhD)
Research Fellow
Tissue Hybridisation & Digital Pathology
Precision Medicine Centre of Excellence
Queen's UniversityBelfast,UK.
Email: M.Sarker@qub.ac.uk
@ Date: 12.01.2021

"""

# # important library installation for train, validate and test the model Detectron2 model
!pip install pyyaml==5.1
# opencv
!pip install opencv-python
# pytorch
!pip install torch==1.7.1+cu101 torchvision==0.8.2+cu101 torchaudio===0.7.2 -f https://download.pytorch.org/whl/
stable.html
import torch, torchvision
print(torch.__version__, torch.cuda.is_available())
!gcc --version
import torch
assert torch.__version__.startswith("1.7")
# detectron2 for CUDA 10.1 + torch 1.7
!pip install detectron2 -f https://dl.fbaipublicfiles.com/detectron2/wheels/cu101/torch1.7/index.html
```

```
In [ ]: # import some common libraries
import warnings
warnings.filterwarnings('ignore')
import torch, torchvision
# You may need to restart your runtime prior to this, to let your installation take effect
# Some basic setup:
# Setup detectron2 logger
import detectron2
from detectron2.utils.logger import setup_logger
setup_logger()
# import some common libraries
import numpy as np
import cv2
import random
# import some common detectron2 utilities
from detectron2 import model_zoo
from detectron2.engine import DefaultPredictor
from detectron2.config import get_cfg
from detectron2.utils.visualizer import Visualizer, ColorMode
from detectron2.data import MetadataCatalog, DatasetCatalog
import detectron2
from detectron2.utils.logger import setup_logger
setup_logger()
```

```
In [ ]: # # convert the ICOS dataset in COCO format and register to detectron2 to train it, this cell only for register the da
taset ( please see the data processing part) :
from detectron2.data.datasets import register_coco_instances
register_coco_instances("icos_train", {}, "icos_train.json", "icos")
register_coco_instances("icos_trainval", {}, "icos_trainval.json", "icos")
register_coco_instances("icos_test", {}, "icos_test.json", "icos")
```

In [ ]:
```
import warnings
warnings.filterwarnings('ignore')
from detectron2.engine import DefaultTrainer
from detectron2.config import get_cfg
import os

## set the configuration for training and validate the model
cfg = get_cfg()

cfg.INPUT.MASK_FORMAT='bitmask'
cfg.merge_from_file(model_zoo.get_config_file("COCO-InstanceSegmentation/mask_rcnn_R_50_FPN_3x.yaml"))
cfg.MODEL.WEIGHTS = model_zoo.get_checkpoint_url("COCO-InstanceSegmentation/mask_rcnn_R_50_FPN_3x.yaml")  # Let traini
ng initialize from model zoo

cfg.DATASETS.TRAIN = ("icos_train",)
cfg.DATASETS.TEST = ("icos_trainval",)
cfg.DATALOADER.NUM_WORKERS = 4

cfg.SOLVER.IMS_PER_BATCH = 4
cfg.SOLVER.OPTIMIZER = 'SGD'
cfg.MODEL.SEM_SEG_HEAD.NORM = "GN"
cfg.MODEL.SEM_SEG_HEAD.LOSS_WEIGHT = 2.0
# Sample size of smallest side by choice or random selection from range give by
cfg.INPUT.MIN_SIZE_TRAIN = (256,)
# Maximum size of the side of the image during training
cfg.INPUT.MAX_SIZE_TRAIN = 256
# Size of the smallest side of the image during testing. Set to zero to disable resize in testing.
cfg.INPUT.MIN_SIZE_TEST = 256
# Maximum size of the side of the image during testing
cfg.INPUT.MAX_SIZE_TEST = 256

cfg.SOLVER.BASE_LR = 0.02  # pick a good LR
cfg.SOLVER.MAX_ITER = 100000    # set the train iterations
cfg.MODEL.ROI_HEADS.BATCH_SIZE_PER_IMAGE = 128   #  (default: 512)
cfg.MODEL.ROI_HEADS.NUM_CLASSES = 1  #  (icos)
cfg.SOLVER.CHECKPOINT_PERIOD = 1000
cfg.SOLVER.GAMMA = 0.1
# The iteration number to decrease learning rate by GAMMA.
cfg.SOLVER.STEPS = (50000, 80000, )
cfg.TEST.EVAL_PERIOD = 1000
cfg.VIS_PERIOD = 1000
```

```
cfg.SEED = 123

## output folder for logdir and model checkpoints
cfg.OUTPUT_DIR = 'Detectron2_ResNet50_B4_SGD_BCE_L1'
os.makedirs(cfg.OUTPUT_DIR, exist_ok=True)
trainer = DefaultTrainer(cfg)
## for training the model (please close this paert during standalone test)
trainer.resume_or_load(resume=True) # for resume the training
trainer.train()
```

In [ ]:

```
## prediction phase
cfg.MODEL.WEIGHTS = os.path.join('Detectron2_ResNet50_B4_SGD_BCE_L1', "model_final.pth")
cfg.MODEL.ROI_HEADS.SCORE_THRESH_TEST = 0.1   # set the testing threshold for this model
cfg.DATASETS.TEST = ("icos_test", )
predictor = DefaultPredictor(cfg)
## getting metadata
icos_metadata = MetadataCatalog.get("icos_test")
dataset_dicts = DatasetCatalog.get("icos_test")
## save the predicted results
import matplotlib.pyplot as plt
%matplotlib inline
from detectron2.utils.visualizer import ColorMode
from torchvision.utils import save_image
from PIL import Image
from pycocotools import mask as mask_util
from detectron2.utils.visualizer import ColorMode
import numpy as np

## save prediction
save_dir = "./Detectron2_ResNet50_B4_SGD_BCE_L1/results/icos/"
s_dir = "./Detectron2_ResNet50_B4_SGD_BCE_L1/results/"
## create dir if not exists
if not os.path.exists(save_dir):
    os.makedirs(save_dir)
## save gt
save_gt = "./Detectron2_ResNet50_B4_SGD_BCE_L1/results/gt/icos/"
save_gt_1 = "./Detectron2_ResNet50_B4_SGD_BCE_L1/results/gt/"
if not os.path.exists(save_gt):
    os.makedirs(save_gt)

for d in random.sample(dataset_dicts, len(dataset_dicts)):
    ## orignal gt
    image_name = d['file_name']
    image_annotations = []
    for annotation in d['annotations']:
        image_annotations.append(annotation)

    segments = [annotation['segmentation'] for annotation in image_annotations]
    ori_masks = mask_util.decode(segments)

    ## predictions
```

```
    im = cv2.imread(d["file_name"])
    ## model predictions
    outputs = predictor(im)
    # print('Number of detected cells:', len(outputs["instances"]))

    v = Visualizer(im[:, :, ::-1],
                   metadata=icos_metadata,
                   scale=1.0,
                   instance_mode=ColorMode.IMAGE_BW   # remove the colors of unsegmented pixels
    )
    v = v.draw_instance_predictions(outputs["instances"].to("cpu"))
    pred_img = v.get_image()[:, :, ::-1]
    # v.save('./instance/'+d["file_name"])

    ## for the predicted mask ( save and visualization )
    mask = outputs["instances"].pred_masks.to("cpu").numpy()
    msks = []
    for m in mask:
      msks.append(m)
    ms = np.sum(msks[:], axis=0)
    ms = np.asarray(ms, np.float)
    m,M = ms.min(), ms.max()
    I = np.asarray((ms - m) / (M - m + 0.000001) * 255, np.uint8)
    I = np.where(I < 1,0,255)
    I = np.asarray(I, dtype=np.int8)
    try:
        ## for saving predictions
        I= Image.fromarray(I).convert('RGB')
        # ## save image and check
        I.save(s_dir+d["file_name"])
        # print(np.unique(I))
#         print(I.size)
        ## for saving GT in same format
        orms = np.sum(ori_masks, -1)
        om = np.asarray(orms, np.float)
        n,N = om.min(), om.max()
        OM = np.asarray((om - n) / (N - n + 0.000001) * 255, np.uint8)
        OM = np.where(OM < 1,0,255)
        OM = np.asarray(OM, dtype=np.int8)
        OM = Image.fromarray(OM).convert('RGB')
        OM.save(save_gt_1+d["file_name"])
#         print(OM.size)
```

```
    except:
        print(I.size)

#     ## predictions visualization
#     fig = plt.figure(figsize=(20,20))
#     ax1 = fig.add_subplot(2,2,1)
#     plt.title(d["file_name"]+'/Number of detected cells:'+ str(len(outputs["instances"])))
#     ax1.imshow(v.get_image()[:, :, ::-1])
#     plt.show()

#     ## GT visualizations
#     # print('Number of original cells:', len(d["annotations"]))
#     visualizer = Visualizer(im[:, :, ::-1], metadata=icos_metadata, scale=1.0)
#     vis = visualizer.draw_dataset_dict(d)
#     ori_image = vis.get_image()[:, :, ::-1]
#     fig = plt.figure(figsize=(20,20))
#     ax2 = fig.add_subplot(2,2,2)
#     plt.title(d["file_name"]+'/Number of original cells:'+str(len(d["annotations"])))
#     ax2.imshow(vis.get_image()[:, :, ::-1])
#     plt.show()
```
